# Reflections on medical student evaluations of a public health clerkship

**DOI:** 10.3389/fpubh.2023.1121206

**Published:** 2023-03-03

**Authors:** Azhar T. Rahma, Balázs Ádám, Aminu S. Abdullahi, Mohamud Sheek-Hussein, Sami Shaban, Mouza AlShamsi, Salama AlKhori, Javaid Nauman, Michal Grivna

**Affiliations:** ^1^Institute of Public Health, College of Medicine and Health Sciences, United Arab Emirates University, Al Ain, United Arab Emirates; ^2^Department of Medical Education, College of Medicine and Health Sciences, United Arab Emirates University, Al Ain, United Arab Emirates; ^3^Department of Statistics, College of Business and Economics, United Arab Emirates University, Al Ain, United Arab Emirates; ^4^Department of Circulation and Medical Imaging, Faculty of Medicine and Health Sciences, Norwegian University of Science and Technology, Trondheim, Norway

**Keywords:** public health, clerkship, reflection, UAE, student evaluations

## Abstract

**Introduction:**

The COVID-19 pandemic demonstrated the need for skilled medical practitioners in public health, and outbreak investigations. The College of Medicine and Health Sciences at the United Arab Emirates University (UAEU) introduced a clerkship in public health constituting theoretical and practical sessions to 5th year medical students in 2015. The aim of this study is to explore the satisfaction of the students with the public health clerkship which is crucial for the assessment and reformation of the taught curriculum.

**Methods:**

A cross-sectional, post-evaluation analysis was conducted from the period 2015–2022. The evaluation questionnaire was conducted *via* an online university system. The survey contained 5 themes: pre-course instructions, structure of the clerkship, academic staff, activities, and learning outcomes. Ethics approval was secured from the Social-IRB of the UAEU. We used SPSS version 26 to analyze the data using independent *t*-test and ANOVA.

**Results:**

One hundred and seventy four students (27.4% response rate) participated in the study. Overall, the students had an average satisfaction score of 2.86 out of 4. The majority of the students reported having a good understanding of public health (93.7%), improving their oral presentation skills (91.2%), and developing new skills (87.2%). Furthermore, more than 9 in 10 students (96.1%) reported that the program expanded their knowledge, skills, and confidence. The mass (90.2%) of students agreed that the clerkship content was covered in sufficient depth, majority of the students agreed that they had received enough information about the clerkship before it started (74.6%), majority of the students agreed that the faculty were interested in their personal development (86.1%) The students who completed the clerkship prior to the COVID-19 pandemic had a statistically significant (*P* = 0.02) higher average rating (72.8%) than students who completed the clerkship during the pandemic (71.1%).

**Discussion:**

Medical students at the UAEU were satisfied with the activities and delivery of the public health clerkship and found it rewarding. Conducting needs assessment and proposal writing provided them with the knowledge, skills, and confidence to conduct research in their career. These findings may be useful in helping and support other institutes to plan and develop a clerkship in the public health.

## Introduction

The overall improvement of the health of the public, not just of individuals, is the main goal of public health medical education. Hence, public health knowledge and competencies are undisputedly a central necessity rather than an option or a mere luxury (1). Historically, the need to include public health, among others, in medical education had been advocated as far back as 1855 (2). However, the importance of basic understanding of public health has again attracted attention nowadays, a development mostly attributable to the recent pandemics including SARS, avian influenza, West Nile Virus and more recently the COVID-19 pandemic (3). Moreover, another notable factor bringing to light the importance of public health in medical education is the current epidemic of chronic non-communicable diseases witnessed in both developed and developing countries (3). The COVID-19 pandemic demonstrated the need for skilled medical practitioners in public health, outbreak investigations and needs assessments. Medical students are the future healthcare taskforce, therefore, training them on community diagnostics and on designing population-based epidemiological studies is fundamental. Fineberg identified six aspects and pillars in which public health matters to medicine, these aspects are: epidemiology; impact of ecological, dietary, societal, and behavioral determinants on wellbeing; systems thinking; culture orientation; population health and global health (4).

The Institute of Public Health (IPH) at the College of Medicine and Health Sciences (CMHS) at the United Arab Emirates University (UAEU) recognized the value of public health and introduced a public health clerkship to 5th year medical students in 2015. The four-week clerkship compulsory public health takes place among the clinical clerkship rotations of main medical specialties during the last 2 years of medical education. Building on the exposure of medical students to biostatistics, epidemiology and health promotion in the 1st and 2nd years, the clerkship provides a unique opportunity to recapitulate public health knowledge and skills, and put them in practical context in the practice-oriented last 2 years of medical training. Such positioning of public health education in a medical program is a quite rare example and provides a recognition of the field among the main medical specialties.

The design of the public health clerkship at the United Arab Emirates University is based on sociocultural theory by the psychologist Lev Vygotsky (5). The sociocultural theory tackles the impacts of society and surroundings on individual education; therefore, in the Institute of Public Health, we adopt a social pedagogy in which students are sensitized to the community approach (6). The clerkship offers a combination of theoretical and practical sessions over a 4-week period 9.00–17.00 daily, except the half-day program on the last working day of the week. The major themes of the consecutive weeks are the main areas of public health, field practice including the field visits, epidemiological study methodology and assessment, respectively. The content spans almost all the major public health themes including biostatistics and basic epidemiology, epidemiology of communicable and non-communicable diseases including injuries, occupational medicine and environmental health, health promotion, health policy and health management. Although theory is discussed in details in lectures, the training focuses on delivering practical public health skills in the form of several case studies, three field visits, portfolios, group-based assessment of public health priorities, and development of epidemiological study proposals. The group-based exercises are typically performed in four groups, each group having a designated faculty supervisor, identifying a separate public health priority based on literature review, and developing a protocol to study the issue. On the last week of the clerkship, students are assessed individually by performing a test of multiple-choice questions and by completing an e-portfolio, as well as in teamwork, when they communicate their findings in the form of oral presentations and written assignments describing the developed protocol.

Student satisfaction is an important aspect of the medical education and exploring the satisfaction of the current medical students with the public health clerkship as well as the impact of the clerkship on their skills is crucial for the assessment and reformation of the taught curriculum. This will allow coordinators and academic staff to reflect on the structure, and activities of the clerkship, and redesign it accordingly. The paper by Jereb et al. associated teaching staff, followed by organizational support, curriculum, physical location, place of the institution, social life and support amenities to student satisfaction (7).

The aim of this study is to explore the satisfaction of medical students with the current structure, content, and activities of the public health clerkship at College of Medicine and Health Sciences at the United Arab Emirates University. Moreover, the study aims at evaluating student satisfaction with the remote delivery of the clerkship during the COVID-19 pandemic.

## Materials and methods

### Sample

The target population of the study was 5th year national medical students who attended the public health clerkship offered by the Institute of Public Health. The medical education department of the college of medicine and health sciences extracted the evaluation of the students over the period of the last seven academic years, from 2015/2016 to 2021/2022.Students. The survey is part of the non-mandatory evaluation of the courses conducted by the Department of Medical Education at the College of Medicine and Health Sciences at the United Arab Emirates University.

### Data collection instrument

The survey is comprised of 37 close-ended and open-ended questions categorized into five themes: pre-course instructions, structure of the clerkship, academic staff, activities of the clerkship including the research project, seminars, and assignments, and learning outcomes. For each statement in the survey, level of agreement could be given on a 4-item Likert scale comprising of “strongly agree,” “agree,” “disagree,” and “strongly disagree.”

### Data analysis

The overall satisfaction and rating of the clerkship program by students was presented as average percentage response scores. These percentage scores were computed by assigning scores of four, three, two and one to the responses strongly agree, agree, disagree, and strongly disagree, respectively, to each of the positive evaluation statements. For the few negative evaluation statements, this order of scoring was reversed to allow for aggregation of overall satisfaction score by each student. The average of these scores was then computed for all the statements and for each student. These averages were then converted to percentage scores to obtain the percentage satisfaction scores. The percentage satisfaction score was tested to assume a skewed distribution using Shapiro–Wilk test for normality. Accordingly, the percentage scores were descriptively summarized using median and interquartile range (IQR). Categorical variables were summarized as frequencies and percentages.

Furthermore, differences in percentage satisfaction scores were examined across gender and period (in relation to COVID-19 to examine the impact of remote teaching and learning on the overall student experience) using Mann-Whitney U test and across academic sessions using Kruskal-Wallis test. Responses to each evaluation statement were also summarized and presented as proportions of the four possible responses—strongly agree, agree, disagree and strongly disagree. Specific responses to all the individual evaluation statements were described and summarized using percentages. Each evaluation statement had four possible ranked responses: strongly agree, agree, disagree, and strongly disagree. Proportions of respondents who indicated any of the options for each evaluation statement were reported alongside the total number of those who responded to the particular evaluation statement. Finally, line graphs were used to illustrate trends in examination score and the clerkship satisfaction score over the academic years. The correlation between these two scores was statistically examined using Pearson correlation coefficient. All inferential statistics were performed with a significance level of 0.05 SPSS version 28 was used for the analysis. (IBM Corp. Released 2021. IBM SPSS Statistics for Windows, Version 28.0. Armonk, NY: IBM Corp).

## Results

A total of 174 clerkship national students of the United Arab Emirates participated in the voluntary evaluation (27.4% response rate), completing a set of questions about their overall clerkship experience. Of these 150 (86.2%) were female and 24 (13.8%) were male. Most participants were from the 2020/2021 academic year (*n* = 32, 18.4%) while the least were during the academic year 2017/2018 (*n* = 16, 9.2%). Table 1 shows the general characteristics of the participants.

**Table 1:** Distribution of students who responded to the clerkship evaluation by gender, academic year, and period with respect to COVID-19 pandemic (*N* = 174).

**Characteristic**	**Frequency**	**Percentage**	**Total number of students**
**Gender**			
Female	150	86.2	482
Male	24	13.8	155
**Academic year**			
2014/2015	26	14.9	80
2015/2016	24	13.8	66
2016/2017	18	10.3	81
2017/2018	16	9.2	97
2018/2019	17	9.8	77
2019/2020	18	10.3	79
2020/2021	32	18.4	73
2021/2022	23	13.2	84
**Period**			
Before COVID-19 pandemic	101	58.0	401
During COVID-19 pandemic	73	42.0	236

Overall, the students had an average satisfaction score of 2.86 out of 4 translating to an average score of71.4% (95% confidence interval = 71.1–73.3). Female students rated the program 72.2% on average, while male students rated it at 70.5% (Table 2). The difference between the two genders was not statistically significant (Mann-Whitney U test, *P* = 0.138). The students who completed the clerkship prior to the COVID-19 pandemic with classroom-based sessions had a statistically significant (Mann-Whitney U test, *P* = 0.02) higher average rating (72.8%) than students who completed the clerkship during the pandemic having online sessions (71.1%), although the difference is not large (Table 2).

**Table 2:** Satisfaction scores by gender, period, and academic year (*N* = 174).

**Characteristic**	**Median (%)**	**IQR (%)**	***P*-value**
**Gender**			
Female	72.2	16.3	0.138
Male	70.5	18.2	
**Period**			
Before COVID-9 pandemic	72.8	15.6	0.020^*^
During COVID-19 pandemic	71.1	15.6	
**Academic year**			
2014/2015	70.8	18.2	0.010^*^
2015/2016	76.9	11.9	
2016/2017	73.6	22.4	
2017/2018	71.1	11.5	
2018/2019	72.8	18.6	
2019/2020	71.7	21.4	
2020/2021	72.8	17.7	
2021/2022	62.8	17.8	

IQR, interquartile range.

^*^P < 0.05.

Statistically significant differences in satisfaction score were also observed across the seven academic years (Kruskal-Wallis test, *P* = 0.01). Students from the 2015/2016 academic year rated the program the highest with an average score of 76.9%, followed by the 2016/2017 academic year with an average score of 73.6%, both of which were pre-COVID-19 pandemic. In contrast, the academic year 2021/2022 (COVID-19 pandemic) had the lowest satisfaction score with a score of 62.8% (Table 2). In general, satisfaction scores for the public health clerkship were typically 0.1–0.4 below the average satisfaction scores for other clerkships delivered on year 5 (Table 3).

**Table 3:** Satisfaction scores for other 5th year clerkships in comparison to the public health clerkship.

**Academic year**	**Average response for public health clerkship (out of 4)**	**Average response for the other 5th year clerkships (out of 4)**
2014/2015	3.30	3.40
2015/2016	3.19	3.60
2016/2017	2.89	3.18
2018/2019	3.21	3.29
2019/2020	2.97	3.34
2020/2021	3.17	3.36
2021/2022	2.82	3.19

**Figure 1:**
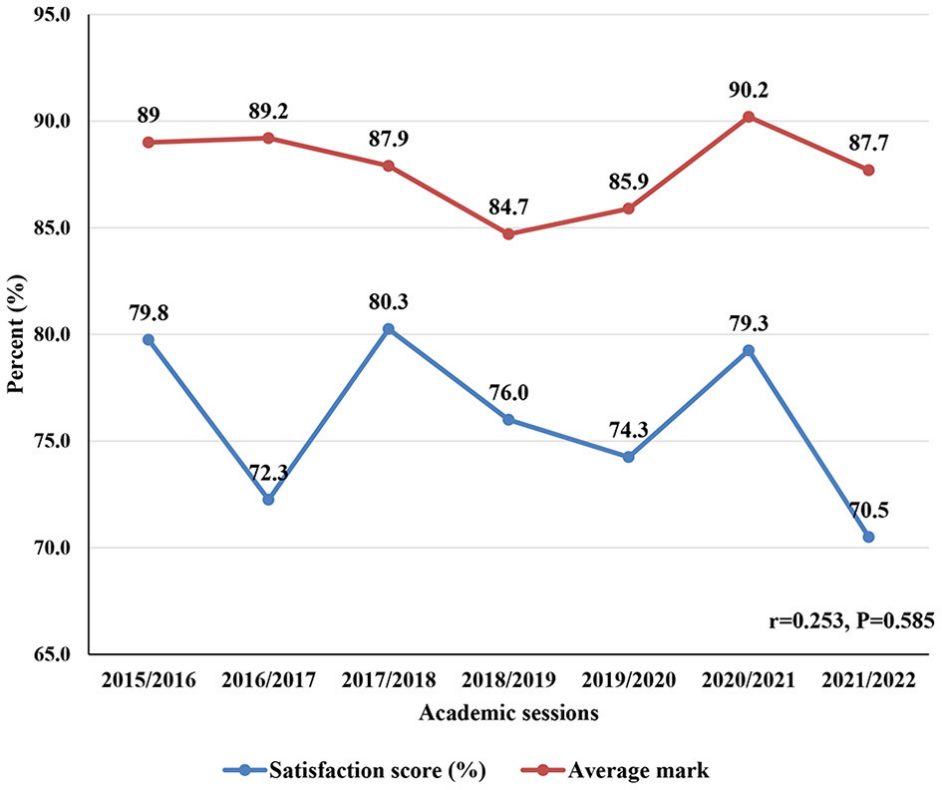
Trends in the public health clerkship satisfaction scores and exam marks.

Figure 1 illustrates the trends in public health clerkship average satisfaction score and the average marks obtained by the students in the clerkship examination. Overall, there was no significant correlation between the scores (*r* = 0.253, *P* = 0.585). However, there seemed to be a positive relationship between the two scores in later academic years of 2019/2020 to 2021/2022 where the average satisfaction score increases or decreases with the corresponding increase or decrease in the average marks obtained in the exam (Figure 1).

Table 4 presents a summary of the students' responses to the specific evaluation statements. Majority of the statements were positive which indicates a positive satisfaction, for instance, “I have found the clerkship rewarding.” Response summary for the few negative evaluation statements are also reported in the table. Overall, a substantial majority (ranging from 72.3 to 96.1%) of the participants agreed to each of the positive statements.

**Table 4:** Summary of students' responses to the specific evaluation statements.

**Statement**	**SA**	**AG**	**DA**	**SD**	**Number of respondents**
**Pre-course instructions**					
I received enough information about the clerkship before it started	31.8	**42.8**	17.9	7.5	173
I received enough information about the assignment	25.7	**53.4**	14.2	6.8	148
I received enough information about the research project	30.6	**50.9**	12.7	5.8	173
I received enough information about the site visit	38.5	**50.0**	7.7	3.8	26
**Structure of the clerkship**					
Overall, the clerkship was well-organized	35.8	**50.9**	8.7	4.6	173
I always knew what was expected of me	28.9	**47.4**	15.0	8.7	173
The clerkship content was covered in sufficient depth	36.4	**53.8**	7.5	2.3	173
The clerkship was a coherent program and NOT just a selection of unrelated subjects	26.0	**54.3**	13.9	5.8	173
There were enough opportunities to ask questions and discuss ideas	37.8	**55.2**	4.1	2.9	172
There was enough time to do a good job	22.7	**55.2**	16.3	5.8	172
There was enough time for reflection	27.9	**51.7**	10.9	9.5	147
Overall, I found self-study sessions useful	31.1	**45.3**	14.2	9.5	148
The self-study sessions gave me an opportunity to clarify issues related to public health	24.5	**51.7**	17.0	6.8	147
I received enough information to be able to self-study	25.7	**52.0**	16.9	5.4	148
The self-study sessions gave me an opportunity to interact with my supervisors	27.9	**49.7**	13.6	8.8	147
The pace of teaching was too fast^*^	**42.3**	19.2	34.6	3.8	26
The pace of teaching was too slow^*^	29.5	31.8	34.7	4	26
There was too much in the timetable^*^	36.6	**38.4**	21.5	3.5	172
There was too little in the timetable^*^	28.0	24.0	**36.0**	12.0	25
The detail in the lectures was just about right	42.3	**46.2**	11.5	0.0	26
Compared with other clerkships the work-load has been heavier*	28.9	**33.5**	31.8	5.8	173
I found the materials on Blackboard useful	34.6	**61.5**	3.8	0.0	26
**Academic staff**					
Teachers were interested in my personal development	35.8	**50.3**	11.6	2.3	173
The assessment has been fair and reasonable	19.7	**56.1**	15.0	9.2	173
The research project assessment has been fair and reasonable	24.0	**64.0**	12.0	0.0	25
The site visit assessment has been fair and reasonable	40.0	**44.0**	12.0	4.0	25
Help was available if I had any problems	28.3	**52.6**	10.4	8.7	173
**Activities of the clerkship**					
Overall, I enjoyed working on the public health assignment and preparing for seminar	27.0	**50.7**	10.1	12.2	148
The public health priority assignment was relevant at this stage in my medical training	23.1	**59.2**	9.5	8.2	147
Overall, I enjoyed the research project	31.2	**48.0**	11.6	9.2	173
The research project was relevant at this stage in my medical training	33.1	**50.6**	12.8	3.5	172
Overall, I enjoyed the site visit	29.5	**46.8**	10.4	13.3	173
The visits were relevant at this stage in my medical training	26.5	**52.4**	9.5	11.6	147
I feel the time would have been better spent on some other activity^*^	**39.3**	38.2	19.1	3.5	173
I didn't like the research project and feel the time would have been better spent on some other activity^*^	**28.9**	28.9	30.6	11.6	173
I would prefer real field research in the community^*^	37.8	**50.7**	6.1	5.4	148
I didn't like the field visits and feel the time would have been better spent on some other activity^*^	25.7	**35.1**	20.3	18.9	148
During the project I always knew what was expected of me	23.1	**69.2**	7.7	0.0	26
Overall, the lectures were relevant at this stage in my medical training	38.5	**53.8**	7.7	0.0	26
**Learning outcomes**					
The clerkship program was relevant at this stage in my medical training	30.1	**50.9**	13.3	5.8	173
I have found the clerkship rewarding	26.0	**50.3**	13.9	9.8	173
I now have a good understanding of what community medicine and public health are all about	43.4	**50.3**	3.5	2.9	173
I feel I need to seek more information on public health issues	17.6	**54.7**	20.9	6.8	148
I feel that I learned a lot and developed new skills	39.2	**48.0**	4.7	8.1	148
I learned data collection techniques	33.8	**51.4**	10.1	4.7	148
I learned data analysis techniques, including descriptive and inferential statistics	33.1	**54.7**	8.1	4.1	148
I learned a lot working on written protocol	34.5	**51.4**	6.1	8.1	148
I improved my oral presentation skills	35.1	**56.1**	4.7	4.1	148
I feel I learnt a lot about public health practice	27.0	**49.3**	12.8	10.8	148
I received enough information about the public health practice	28.4	**56.1**	5.4	10.1	148
I learned about the infectious disease screening process	35.8	**48.6**	4.7	10.8	148
I learned about preventive services	34.5	**50.7**	4.7	10.1	148
I learned about health promotion programs	31.1	**52.0**	7.4	9.5	148
I learned about electronic notification	35.8	**49.3**	4.7	10.1	148
The project has encouraged me to do research in the future	34.6	**61.5**	3.8	0.0	26
I am satisfied with my achievements in this rotation	42.3	**53.8**	3.8	0.0	26
This clerkship rotation has expanded my knowledge, skills, and confidence	40.0	**56.0**	4.0	0.0	25

SA, Strongly agree; AG, Agree; DA, Disagree;SD, Strongly Disagree.

^*^Negative evaluation statements. Bold values indicates the highest proportion.

### Pre-course instructions

The majority of the students agreed that they had received enough information about the clerkship before it started (74.6%), about the assignments (79.1%), about the research project (81.5%), as well as about the site visit (88.5%) (Table 4).

### Structure of the clerkship

About 90% of the students agreed that the clerkship content was covered in sufficient depth, and that 93% agreed there were enough opportunities to ask questions and discuss ideas. However, the majority of them also lamented that there was too much in the program timetable (75%) and that the workload of the clerkship was heavier when compared with the other clerkships (62.4%) (Table 4). Below are some opinions of the students:

“*The self study session was a really good session to benefit from.”*

“*I would like to have critical thinking session able the student to think about a solution of public health issues.”*

“*Too much works to do and too much heavy subjects that I think I don't need it in my future either*.”

### Academic staff

The students were quite pleased with the faculty members throughout the course as assessed by five statements in the evaluation. The majority of the students agreed that the faculty were interested in their personal development (86.1%), the assessment by the faculty was fair and reasonable (84%), and that the faculty members were ready to help in case of any problems (80.9%) (Table 4). A comment on the strengths of the clerkship is:

“*Very helpful and kind tutors. They like their field which was reflected by the way they teach us. Great!”*

### Activities of the clerkship

Overall, the majority of students indicated that they are happy with the various activities, including research and practical sessions. However, while the majority of students agreed that they found the public health priority assignment (82.3%), the research project (83.7%), and the site visits (78.9%) relevant, the majority (88.5%) of students reported that they would prefer real field research in the community (Table 4). Below are some comments on strengths of the clerkship:

“*The self- study sessions helped us improve our data collection skills.”*

“*Research project was my favorite part of the whole rotation. learned a lot from it.”*

“*The visits were relevant at this stage in my medical training.”*

### Learning outcomes

The majority of students rated the clerkship program favorably regarding its learning outcomes. They had a good understanding of what community medicine and public health were all about (93.7%), they improved their oral presentation skills (91.2%), and they developed new skills (87.2%). Furthermore, 96.1% of students indicated that they were satisfied with their achievements in the clerkship and that the program expanded their knowledge, skills and confidence (Table 4). Below are some of the comments of the students about the learning outcomes:

“*The protocol benefited us as students because it taught us how writing a research looks like and how is information gathered. Nevertheless, the doctors were helpful.”*

“*The project helped me understand the concepts that were taught during the sessions and apply the knowledge in depth. I appreciate this and am really thankful for it.”*

“*The rotation would be best if it were given at an earlier stage in our medical school. for example at the premed or preclinical stage. Since we were given the opportunity to learn how to write a research protocol, and many students would like to conduct different types of research earlier and this would help them vastly.”*

“*Overall, the Public Health rotation opened my eyes to understand the health needs of my society and how to approach it. The group work and addressing critical issues as a team really helped my personal and academic development.”*

## Discussion

The results of this study show that medical students at the United Arab Emirates University were satisfied with the activities and delivery of the public health clerkship and found it rewarding. Conducting needs assessment and proposal writing provided students with the knowledge, skills, and confidence to conduct research in their career.

The majority of our cohort were females (86.2%) and that mirrors the common ratio of females in medical schools in the UAE (8, 9), and yet there was no statistically significant difference in satisfaction level between genders in our cohort. Our study revealed that the medical students were less satisfied with the clerkship during COVID-19 pandemic in comparison with their pre-pandemic satisfaction. We articulate that the students wanted the social interaction with peers in addition to attending field and site visits (to occupational medical clinic, Public Health Center and the Environment Agency), which were on hold during the pandemic. Our findings are in consonance with the literature, a nationwide cross-sectional study of 2,721 UK medical students conducted by Dost et al. in May 2020 across 40 UK medical schools alluded those medical students did not find online teaching to be engaging or enjoyable, with limited chances to raise queries. Besides, they did not find it as effective as face-to-face teaching (10). These findings are parallel to our results, since our medical students were less satisfied with the field visits, especially during the COVID-19 pandemic when all sessions were provided online, including “virtual visits,” during which invited field practitioners explained public health activities but without real hands-on practical engagement. Another approach to interpret these results is that because of the pandemic, everyone—including medical students—had much greater awareness of public health/global health problems and efforts to ameliorate the conditions, and thus went into the course with a greater understanding of public health and a greater expectation of what they would learn from the course.

. While studying the impacts of society and surroundings on individual education, the sociocultural theory explains that a student's psychological development is steered by mentors and teachers as well as communication with social assembles (5). This can be translated by the satisfaction with the activities of the public health clerkship and their expressed preference of the real field research in the community (88.5%).

The pedagogies that are rooted in the public health clerkship offered by the Institute of Public Health are a mixture of active learning, project-based learning, cooperative learning and context-based learning (11). The hybrid of pedagogies addressing the different learning styles of the students allows faculty to engage students and apply concepts as well as steering students to work together to exploit their own and each other's learning journey. A randomized controlled trial conducted to test the power of pedagogies on learning outcomes in public health concluded that cooperative learning pedagogy improved the performance on higher cognition in contrast to the self-study pedagogy, a technique that is used throughout our public health clerkship (12). Cooperative learning tackles cooperative skills therefore public health clerkship emphasized the value of group work by dividing the students to groups from day one to produce a group protocol and present it as a group to the faculty and their peers. The group work helps prepare the students for the practice in healthcare setting as they will eventually work as a team with other healthcare providers.

The structure of the clerkship offered by the Institute of Public Health bridges all the four P's: Public health, Prevention, Population health, and Policy (13). The themes include all the information fundamental to public health, namely: Biostatistics, Epidemiology, Environmental Health Sciences, Health Services Administration, Social and Behavioral Sciences. These are core accreditation areas used by accrediting bodies including the Council on Education for Public Health (CEPH) in the USA (14). A scoping review of studies evaluating the education of health professional students about public health conducted by Evashwick et al. acknowledged a dearth of the literature on appraisals of methodologies for teaching public health (15). The medical students were satisfied with the structure of the clerkship and stated that it was well-organized, and that it was coherent and not just a selection of unrelated subjects. They embraced the structure of the clerkship in terms of self-study sessions and opportunities to interact with their supervisors as well as the easiness of retrieving materials on the learning management system (Blackboard).

The students stated that teachers were interested in their personal development and were available if the students had any problems. In each public health clerkship at the Institute of Public Health, students are divided into groups and assigned a mentor from day one. The mentors support the students in conducting the needs assessment and proposal writing. The timetable of the clerkship has scheduled sessions for the students to meet with their mentors on daily basis. The mentors oversee the students' progress, address their challenges, provide them with resources and introduce them to stakeholders. The mentors help the students navigate and select pressing public health issues that are of value to the United Arab Emirates. In addition to the development of a study protocol, the mentors may secure funding to the students by applying for the internal grant named the Summer Undergraduate Research Experience (SURE) PLUS to explore and test a research idea and to promote and support the engagement of undergraduate students in research experiences, thereby providing the students with exposure to, and training in, conducting research while working in teams under the supervision of qualified faculty members. Moreover, all abstracts of the students' proposals are published in a book of abstracts (16). It is worth mentioning that in addition to the assigned mentor, other faculty can provide additional mentorship to the students based on their area of expertise and research interests. These practices are in synch with the literature investigating the effect of mentorship in the success and academic development of the mentees, especially in the Science, Technology, Engineering and Mathematics (STEM) fields (17, 18).

As expected with the subjective nature of survey, students varied in their responses toward pace of teaching and the timetable as some (75%) stated that there was too much in the timetable. In contrast 52% stated in another evaluation statement that there was too little in the timetable. Variable expectations among medical students are documented in the literature. A qualitative exploratory study conducted by Yoon et al. in 2021 among medical students in dermatology clerkship echoed a variability in the student's experiences (19).

Medical students in the United Arab Emirates University are eager for literacy in public health and that desire was amplified during the pandemic. In our cohort, all questions related to learning outcomes were on the agreement side. Students found the clerkship rewarding and acquired a good understanding of the value of public health for the community. Students were satisfied with the soft skills that they fostered, like oral presentation skills and preparation for seminars. These learning outcomes are in line with the Institute of Medicine's (IOM) radical report “The Future of Public Health “that reformed and revolutionized the public health education and called for equipping public health practitioners with salient methodological, political, administrative and programmatic skills (20).

The strength of our study is being the first to address the satisfaction of undergraduate medical students with regard to the public health clerkship in the UAE. The study covered a wide scope of themes including pre-course instructions, structure of the clerkship, the academic staff, activities of the clerkship and learning outcomes. The hybrid of open-ended and close questions allowed the detailed investigation and analysis of students' responses. These findings can support other medical colleges and institutions establishing or reviewing a public health clerkship. Nevertheless, since the questions on student satisfaction asked by the Department of Medical Education are standardized, and therefore fixed, we were unable to diagnose further details of some issues, which warrant further investigation. The inherited biases of cross-sectional surveys constitute a limitation of this study. The low response rate is another limitation that we faced, but due to the voluntary and anonymous nature of the feedback centrally organized by the College, we could not control. That was one of the reasons why we included several academic years to have a better understanding of the students' satisfaction. Response rate varied over the years and female medical students were significantly more likely than males to complete the evaluation form. Moreover, the low response to the open-ended questions deprived this analysis from in-depth understanding of students' opinions. A very long student feedback form is used. It might have had a larger response if it was briefer. The anonymous nature of the survey hindered connecting satisfaction to academic achievement and other characteristics on individual level. Moreover, not having a tailored survey for students' satisfaction during the pandemic limited our understanding of their poorer satisfaction in this period. The lack of records at the University about the specializations chosen after graduation is another limitation that made linking our findings to the future carrier path of the students impossible.

Our findings warrant further investigations that may include conducting a qualitative focus group discussion among alumni. Such studies will reveal the impact of this clerkship on their career path, and by that help to improve the curriculum to better meet the needs of public health practice, and increase its satisfaction scores that are typically lower than for the other clerkships.

In conclusion, the public health clerkship offered by the Institute of Public Health at the College of Medicine and Health Sciences at the United Arab Emirates University facilitated a good understanding of what community medicine and public health were all about and improved the oral presentation skills of the fifth-year medical students. Furthermore, majority of the students indicated that they were satisfied with their achievements in the clerkship and that the program expanded their knowledge, skills, and confidence. If there is a need for an online offering of the clerkship, the structure of the rotation needs to be reformed. The results of this study can be utilized for the further development of the clerkship curriculum with the final aim to provide the necessary basic public health knowledge and skills for all graduating medical doctors.

## Data availability statement

The original contributions presented in the study are included in the article/supplementary material, further inquiries can be directed to the corresponding author/s.

## Ethics statement

The studies involving human participants were reviewed and approved by Social-IRB of the UAEU. Written informed consent for participation was not required for this study in accordance with the national legislation and the institutional requirements.

## Author contributions

AR, BÁ, MG, and SS conceived the study. AA, MA, and SA acquired and analyzed the data. AR and AA produced the first draft. BÁ, MG, MS-H, and JN reviewed the first draft. All others reviewed the final draft.
